# *Botulinum Neurotoxin* A Injections Influence Stretching of the Gastrocnemius Muscle-Tendon Unit in an Animal Model

**DOI:** 10.3390/toxins4080605

**Published:** 2012-08-13

**Authors:** Patrick Haubruck, Sandeep Mannava, Johannes F. Plate, Michael F. Callahan, Walter F. Wiggins, Gerhard Schmidmaier, Christopher J. Tuohy, Katherine R. Saul, Thomas L. Smith

**Affiliations:** 1 UniversitätsKlinikum Heidelberg, Stiftung Orthopädische Universitätsklinik, Schlierbacher Landstrasse 200a, Heidelberg, 69118, Germany; Email: Gerhard.schmidmaier@med.uni-heidelberg.de; 2 Department of Orthopaedic Surgery, Wake Forest School of Medicine, Medical Center Boulevard, Winston-Salem, NC 27157, USA; Email: smannava@wakehealth.edu (S.M.); callahan@wakehealth.edu (M.F.C.); wwiggins@wakehealth.edu (W.F.W.); ctuohy@wakehealth.edu (C.J.T.); tsmith@wakehealth.edu (T.L.S.); 3 The Neuroscience Program, Wake Forest University Graduate School of Arts and Sciences, Medical Center Boulevard, Winston-Salem, NC 27157, USA; 4 Department of Biomedical Engineering, Wake Forest School of Medicine and VT-WFU School of Biomedical Engineering and Sciences, Medical Center Boulevard, Winston-Salem, NC 27157, USA; Email: ksaul@wakehealth.edu

**Keywords:** *Botulinum Neurotoxin*, spasticity treatment, muscle tone, passive muscle biomechanics

## Abstract

*Botulinum Neurotoxin* A (BoNT-A) injections have been used for the treatment of muscle contractures and spasticity. This study assessed the influence of (BoNT-A) injections on passive biomechanical properties of the muscle-tendon unit. Mousegastrocnemius muscle (GC) was injected with BoNT-A (*n* = 18) or normal saline (*n* = 18) and passive, non-destructive, *in vivo* load relaxation experimentation was performed to examine how the muscle-tendon unit behaves after chemical denervation with BoNT-A. Injection of BoNT-A impaired passive muscle recovery (15% *vs.* 35% recovery to pre-stretching baseline, *p* < 0.05) and decreased GC stiffness (0.531 ± 0.061 N/mm *vs.* 0.780 ± 0.037 N/mm, *p* < 0.05) compared to saline controls. The successful use of BoNT-A injections as an adjunct to physical therapy may be in part attributed to the disruption of the stretch reflex; thereby modulating *in vivo* passive muscle properties. However, it is also possible that BoNT-A injection may alter the structure of skeletal muscle; thus modulating the *in vivo* passive biomechanical properties of the muscle-tendon unit.

## 1. Introduction

Spasticity is a debilitating and complex hyperkinetic syndrome characterized by excessive excitability of the stretch reflex (myotatic reflex) and loss of neuronal control leading to muscle overactivity with the inability of the muscle to rest [1,2,3]. In post-stroke patients, spasticity affects the extremities, with clinical impairment in up to 40% of patients [4]. Cerebral palsy is caused by injury to the developing central nervous system, often resulting in reduced health-related quality of life [5,6,7,8,9]. Lesions to upper motor neurons, regardless of etiology or mechanism, affect the ability of the central nervous system to synchronize motor unit recruitment and to relax skeletal muscle [2]. Activation of the peripheral stretch reflex alters resting posture of the affected limb and impairs passive function [2], which leads to structural muscle changes over time that increase muscle stiffness and contribute to the clinical symptoms of spasticity [10].

Physical therapy and rehabilitation of patients with spasticity involves stretching of stiff muscle-tendon units that resist manipulation. Stretching has been shown to influence skeletal muscle by altering active contractility, passive biomechanical properties, and neural excitability [11]. Previous studies have documented decreased stiffness [12,13,14], improved movement control [15], and fewer contractures [16] after therapeutic stretching interventions in spastic patients. 

BoNT-A application as an adjunct to physical therapy in patients with spasticity has been shown to reduce pain, improve gait pattern, and improve range of motion [5]. The rationale for the use of BoNT-A injections into spastic muscle is to decrease muscle overactivity and correct postural deformity [5]. BoNT-A is endocytosed by the distal neuron and prevents the pre-synaptic vesicular fusion mediated by SNARE proteins (synaptobrevin, syntaxin, and synaptosomal-associated protein-25) and thus the release of acetylcholine at the neuromuscular junction inhibiting normal synaptic transmission and neural-mediated muscle contraction [5]. Injection of BoNT-A induces a temporary and reversible paresis of skeletal muscle with a decrease of motor action potential (CMAP) of up to 80% one week after injection depending on dose and volume applied [17]. The adjunctive application of BoNT-A to physical therapy of patients with muscle spasticity has been shown to improve clinical outcomes and decrease symptoms [5]. 

While BoNT-A has been used clinically as an effective adjunct to physical therapy in patients with spasticity [5], limited basic science studies have examined the influence of BoNT-A on the *in vivo* passive properties of skeletal muscle and the influence of BoNT-A injection on the myotatic or stretch reflex. Animal models of spasticity with experimental induction of an upper motor neuron lesion are not well established, and those spastic disease animal model systems that have been described are inherently variable as spastic disease states have pathologic myotatic or stretch reflexes [18,19]. The use of healthy, non-spastic muscle to study effects of BoNT-A on *in vivo* biomechanical properties isolates the effects of BoNT-A without the variability inherent in spastic disease. The purpose of this study was to investigate the influence of BoNT-A injection on passive biomechanical muscle properties in normal mouse gastrocnemius (GC) muscle in response to repetitive, non-traumatic stretching. This study hypothesized that BoNT-A modulates *in vivo* skeletal muscle tone and disrupts the myotatic or stretch reflex, thereby decreasing muscle stiffness during stretching.

## 2. Materials and Methods

Thirty-six male CD1 mice weighing 25.56 g ± 0.64 g (Charles River, Wilmington, MA) were used for this study. The experimental protocol was approved by the Institutional Animal Care and Use Committee in accordance with the standards required by the National Institutes of Health. Animals were kept in a controlled temperature vivarium with a 12-h light/dark cycle, with food and water provided *ad libitum*.

Animals were randomly assigned to two experimental groups; the study group (*n* = 18) received BoNT-A at a dose of 6 U/kg in a volume of 10 µL into the GC muscle, the control group (*n* = 18) received an equal volume of normal saline solution (0.9%). Volume and dose of BoNT-A were chosen to provide maximal denervation, and all biomechanical assessments were performed eight days following injection to reach maximal denervation based upon previous dose-volume experimentation in our laboratory using this animal model system [17,20].

### 2.1. Botulinum Toxin A and Saline Injection

The animals were anesthetized with 1.5–2.0 vol.% isoflurane (Webster Veterinary, Patterson Companies Inc., Sterling, MA) during the injection procedure. The reconstitution of BoNT-A (Allergan, Irvine, CA) and the percutaneous gastrocnemius-soleus injection were performed using previously described methods [17,20,21]. Normal saline solution was injected in the same manner using the same volume as the toxin injection for control animals.

### 2.2. Surgical Approach and Experimental Set Up

The experimental apparatus and the surgical approach were similar to previous reports [21,22]. Briefly, eight days post-injection, the saline-injected control animals (*n* = 18) and BoNT-A experimental animals (*n* = 18) were anesthetized with isoflurane (1.5–2.0 vol.%). The animals were then placed on a linear translating stage and secured with a body strap and Kirschner wires through the knee, foot, and adjacent soft tissue to eliminate movement artifact. The calcaneus was transected, the soleus muscle dissected from its calcaneal insertion, and the gastrocnemius-Achilles-calcaneus (muscle-tendon-bone) unit connected to a force transducer (model FORT100; World Precision Instruments Inc., Sarasota, FL) with stainless steel suture. The force transducer was connected to an amplifier (model ps100W-2, EMKA Technologies Inc., Falls Church, VA) through an interface controller (EMKA Technologies Inc., Falls Church, VA) and connected through an analog-to-digital converter card (model PCI-6023E, National Instruments, Austin, TX) to a personal computer. 

For *in vivo* biomechanical testing, the sciatic nerve was exposed and stimulated with a bipolar hook electrode (FHC, Bowdoinham, ME) connected to a pulse generator (model Tenma TGP110, BioSurplus Inc., San Diego, CA). The force of muscle contraction and the passive muscle-tendon unit properties were recorded continuously (IOX software, Slow Wave Analyzer, EMKA Technologies Inc., Falls Church, VA). A heat lamp was used during the entire experimental protocol to maintain animal body temperature.

### 2.3. Muscle Force Assessment/Load-Relaxation Assessment

For each animal, a range of stimuli from 0.8 V to 1.6 V was delivered to the sciatic nerve in order to determine the supra-maximal voltage required to produce maximal single-twitch contraction. In all instances, 1.2 V was used during the active force of contraction protocol [20,21,22]. For tetanic contraction force, a square wave stimulus of 1.2 V at a frequency of 100 Hz for 1 s was used. A recovery time of 30 s was allowed between stimuli [21]. Muscle force assessment was conducted to verify that the BoNT-A injection was effective in decreasing active muscle force of single twitch and tetanic contraction. 

After the active muscle force testing protocol was completed, a muscle preconditioning protocol was used prior to passive load-relaxation testing with a strain rate of 0.6 mm/s for all experiments. The muscle-tendon unit is visco-elastic tissue; therefore it was necessary to continuously record the developed passive tension after a displacement, since the tissue would relax in response to a stress. Three non-destructive *in vivo* stretching protocols were utilized to examine the passive, biomechanical muscle-tendon unit response to BoNT-A and saline injection.

#### 2.3.1. Protocol 1

The muscle-tendon unit was stretched from a baseline displacement of 0mm to displacements of 1 mm, 2 mm, and 4 mm. Passive tension was continuously recorded for 210 s at each displacement interval. Between loading cycles, the muscle-tendon unit was returned to 0 mm displacement and allowed to recover for 180 s. Protocol 1 was designed to investigate differences in the *in vivo* stiffness of the chemically denervated muscle-tendon unit, when compared to saline-injected controls. This investigation was based upon a linear-regression analysis of the *in vivo* linear portion of the passive tension versus displacement curve. This method expands upon a previously published method in the mouse GC muscle [21]; however, it includes a more thorough biomechanical consideration of the visco-elastic tissue, in that both peak and equilibrium stiffness were examined.

#### 2.3.2. Protocol 2

The muscle-tendon unit was stretched from 0 mm to 4 mm, while passive tension was continuously recorded for 210 s. Then, the muscle-tendon unit was returned to 0 mm displacement and allowed to recover for 180 s. The entire stretching cycle (4 mm displacement returning to 0 mm baseline displacement) was repeated for four cycles. This protocol was designed to investigate differences in biomechanical behavior of denervated muscle and normal muscle during repeated stretching.

#### 2.3.3. Protocol 3

The muscle-tendon unit was stretched from 0 mm to 1 mm of displacement and passive force was continuously recorded for 240 s. The passive force generated at the initial 1 mm displacement, prior to the rest of the stretching protocol being performed, was used as the “baseline” passive tension for the muscle-tendon unit at a length of 1 mm. Then, the muscle-tendon unit was stretched from a baseline displacement of 0 mm to 4 mm of displacement, where it was held for 120 s. The muscle-tendon unit then was returned to a “displacement” of 1 mm, and passive tension was continuously recorded for 240 s. This protocol was designed to study the recovery of passive tension in the muscle-tendon unit after it returned to a shorter length after being held in a stretched position.

To evaluate the *in vivo* stiffness and the effects of stretching on the passive tension of the muscle-tendon unit, experimentation was conducted in the linear portion of the stress-strain curve. The muscle-tendon unit was displaced to a range of 0 mm, 1 mm, 2 mm, and 4 mm to avoid gross-injury to the muscle-tendon unit. This range of displacements avoided the failure region or yield-region of the muscle-tendon unit stress-strain curve [21]. 

### 2.4. Statistical Analysis

All results are expressed as mean ± standard error of the mean. Student t-tests were used to analyze the results of muscle force testing (e.g., maximal single twitch and tetany twitch). For the results of the load-relaxation assessment (peak tension, equilibrium tension and visco-elastic parameters), a mixed model ANOVA followed by a Tukey post-hoc test was performed. A linear regression analysis was performed on the elastic peak tension and equilibrium tension versus time. All statistical tests were performed using SigmaStat (Systat Software, Inc., San Diego, CA). 

## 3. Results

**Figure 1:**
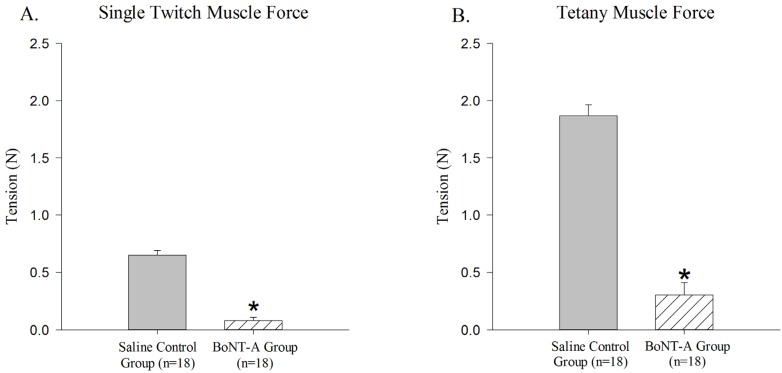
Reduction of active force produced in *Botulinum Neurotoxin* A (BoNT-A) injected gastrocnemius muscle (GC) muscle compared to saline injected control muscle. BoNT-A injected muscle produces significantly less force in both single twitch and tetanic contraction (*p*<0.05). (**A**) Maximal force produced in a single twitch muscle contraction 1 week after BoNT-A injection. (**B**) Maximal force produced in tetanic muscle contraction 1 week after injection. Data are presented as the mean and standard error of the mean.

The BoNT-A injected GC muscle produced less active single-twitch (*p* < 0.05, Figure 1A) and tetanic contraction force (*p* < 0.05, Figure 1B) when compared to the saline-injected control group. The BoNT-A injected group produced an average of 0.079 N ± 0.03 N for single twitch force and an average of 0.302 N ± 0.108 N for tetanic contraction force. The saline injected group produced an average of 0.651 N ± 0.038 N for single twitch force and an average of 1.87 N ± 0.095 N for tetanic force. 

**Figure 2:**
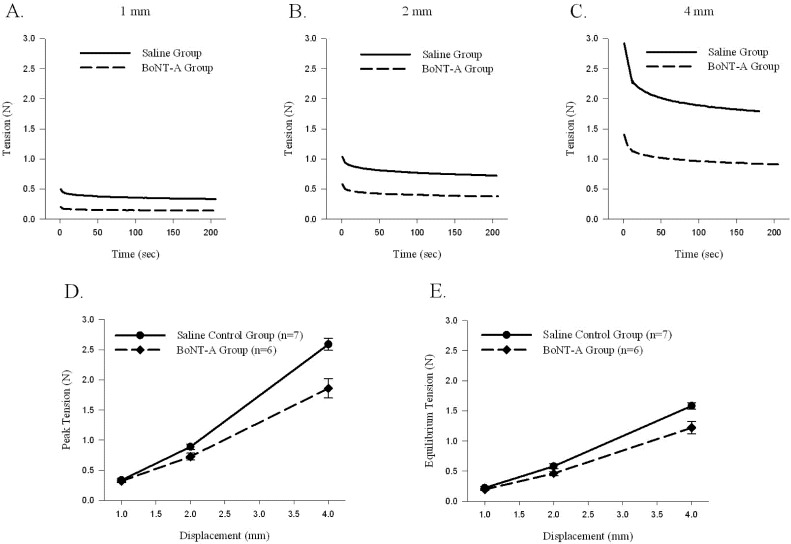
The effect of displacement (1, 2 and 4mm) on passive peak and equilibrium tension produced in the muscle-tendon unit. Peak and equilibrium tension produced by the muscle-tendon unit increases with rising displacement; tension produced by the BoNT-A injected muscle-tendon unit is significantly lower during 4mm of displacement (*p*<0.05). (**A**–**C**) Sample time histories of passive force produced by stretching BoNT-A injected muscle-tendon unit and saline control muscle-tendon unit after displacements of 1, 2 and 4mm, respectively. (**D**) Average passive peak tension produced during various displacements. (**E**) Average passive equilibrium tension produced during various displacements. Data are presented as the mean and standard error of the mean.

Sample time histories for muscle-tendon unit displacements to 1 mm (Figure 2A), 2 mm (Figure 2B), and 4 mm (Figure 2C) during Protocol 1 showed that the BoNT-A injected muscle-tendon unit generated less passive tension throughout the displacement protocol. When compared to the saline-injected controls, the BoNT-A injected experimental group produced less average peak tension (*p* < 0.05, Figure 2D) and less average equilibrium tension (*p* < 0.05, Figure 2E). The average muscle-tendon unit peak stiffness of the BoNT-A injected group was 0.520 ± 0.06 N/mm, compared to the saline injected group, which was 0.764 ± 0.036 N/mm, representing 31.9% decline of *in vivo* stiffness one-week following chemical denervation with BoNT-A (*p* < 0.05, Table 1). The average muscle-tendon unit equilibrium stiffness of the BoNT-A injected group was 0.346 ± 0.038 N/mm, compared to the saline injected group, which was 0.459 ± 0.012 N/mm, representing 24.6% decline of *in vivo* stiffness one-week following chemical denervation with BoNT-A (*p* < 0.05, Table 1).

**Table 1:** Linear regression analysis for muscle-tendon unit displacement *versus* passive tension.

	Slope (N/mm)	*R* ^2^	*P*
**Peak Tension**			
Saline Control Group ( *n* = 7)	0.764 ± 0.036	0.990	0.059
BoNT-A Group ( *n* = 6)	0.520 ± 0.06 *	0.992	0.046
**Equilibrium Tension**			
Saline Control Group ( *n* = 7)	0.459 ± 0.012	0.992	0.012
BoNT-A Group ( *n* = 6)	0.346 ± 0.038 *	0.991	0.048

Linear regression analysis performed on peak passive tension *vs.* displacement data (Figure 2D) and equilibrium passive tension *vs.* displacement data (Figure 2E) in accordance with previously published analysis [21]. All data expressed as mean ± standard error of the mean. * *p* < 0.05.

**Figure 3:**
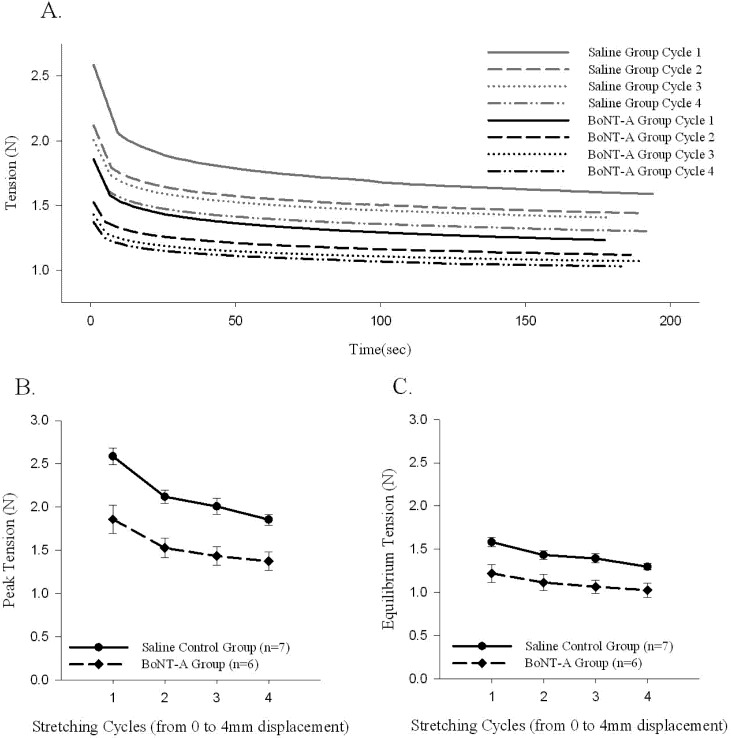
The effect of repeated stretching on passive peak and equilibrium tension produced in the muscle-tendon unit. Repeated stretching of the muscle to 4mm decreases the passive peak and equilibrium tension produced in each cycle. Both peak (*p*<0.05) and equilibrium (*p*<0.05) tension produced in the denervated muscle-tendon unit is significantly lower during all cycles when compared to control muscle. (**A**) Sample time histories of passive tension produced in both groups during each 4 mm displacement cycle; both groups trend to a stable level after repeated stretching and the BoNT-A injected muscle tension unit produces less force in each cycle. (**B**) Average passive peak tension produced during repeated stretching. (**C**) Average passive equilibrium tension produced during repeated stretching. Data are presented as the mean and standard error of the mean.

A sample time history of passive force generated and the stress-relaxation behavior of the muscle-tendon unit after repeated stretching to 4 mm of displacement (Protocol 2) is shown (Figure 3A). The initial generation of peak tension, followed by a relaxation of the passive generated force to an equilibrium tension over the time course of the experiment, was observed in all stretched muscle-tendon units (Protocol 2, Figure 3A). Repeated stretching of the muscle tissue resulted in a declining average passive force throughout the time history after each cycle of stretching (Figure 3). The BoNT-A injected muscles generated less passive tension after stretching (Protocol 2), when compared to saline-injected controls, throughout the time history (Figure 3A, *p* < 0.05), for peak tension (Figure 3B, *p* < 0.05), and for equilibrium tension (Figure 3C, *p* < 0.05). The BoNT-A group generated less passive tension throughout the stress-relaxation period (Protocol 2), regardless of cycle number, when compared to the saline injected controls at each cycle (Figure 3).

**Figure 4:**
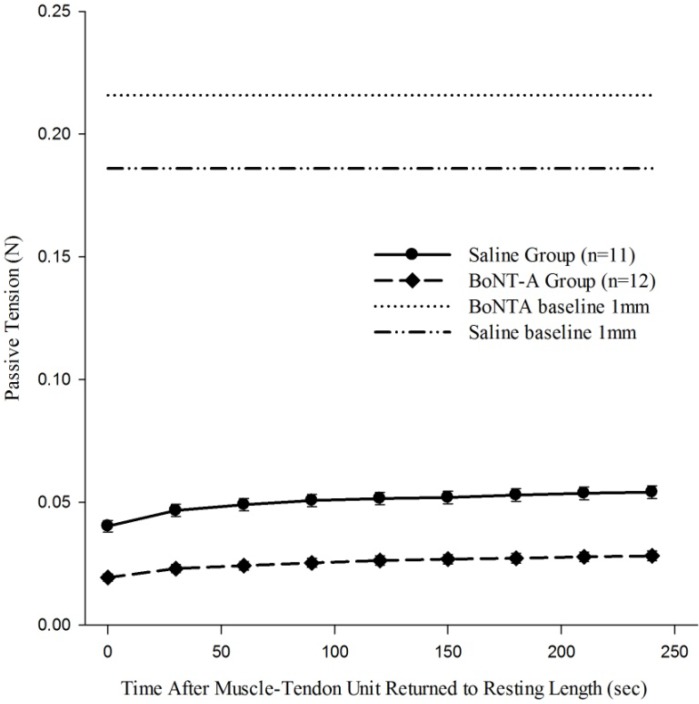
The effect of chemical denervation on the recovery of passive tension after return to a shorter length of 1mm. Passive tension produced after the muscle-tendon unit was returned from 4mm of displacement to 1mm of displacement was significantly lower throughout the time history for the chemically denervated muscle-tendon unit (*p*<0.05). Data are presented as the mean and standard error of the mean.

When the recovery of passive tension in the muscle-tendon unit was evaluated after it was returned to 1 mm baseline length after being held in a stretched 4 mm displacement (Protocol 3), the muscle regained some passive tone as indicated by increased passive tension generated as time increases (Figure 4). The BoNT-A injected group had reduced passive tension (*p* < 0.05, Figure 4) compared to the saline-injected group at all time points. Further, BoNT-A impaired the passive recovery of the muscle-tendon unit after it was returned to initial length; the BoNT-A injected group only recovered 15% of the pre-stretching baseline tension, while the saline injected group recovered 35% of the pre-stretching baseline tension (*p* < 0.05, Figure 4).

## 4. Discussion

BoNT-A injection affected the passive soft-tissue properties resulting in significantly reduced *in vivo* peak and equilibrium muscle stiffness compared to controls during repeated stretching. Injected muscle was more compliant compared to saline controls. In particular, BoNT-A injections reduced *in vivo* stiffness of the muscle-tendon unit by approximately 30%, which is similar to previous findings [21,22]. In these reports, the authors suggested that BoNT-A injection might improve the excursion of the muscle-tendon unit by modulating stiffness [21,22]. Based upon the findings of the current study, BoNT-A has a favorable effect on skeletal muscle during repeated stretching, improving the compliance of the muscle-tendon unit and altering resting muscle tone, ultimately allowing for greater relaxation following stretching. The present study expands upon previous work by specifically examining the influence of BoNT-A injection during *in vivo* stretching of muscle-tendon units and the myotatic or stretch reflex.

The effect of BoNT-A on passive properties of skeletal muscle may be mediated through different mechanisms. The quantification of the central nervous system’s influence on *in vivo* soft-tissue biomechanical properties has not been extensively studied, despite the clinical knowledge that individuals with nervous system dysfunction undergo passive property changes of their muscle-tendon units [3,5,6,7,8,9]. Historically, passive muscle properties have been attributed to structural elements of the muscle-tendon unit, such as connective tissue, contractile components (e.g., actin, myosin), and intracellular components (e.g., titin, desmin) [23]. Leonard and Herzog performed single myofibril experiments in which passive force enhancement after stretching was observed following maximal activation and complete stretching past actin-myosin cross-bridging. Based upon their results, the authors postulated that the protein titin, not just actin-myosin cross-bridging, likely contribute to the passive force enhancement observed upon returning to a shorter displacement after stretching [24]. Many published reports have studied the individual structural components that contribute to the passive tension generated by a muscle-tendon unit after stretching; however, many of these studies were limited by *in vitro* or *ex vivo* experimental settings and assessment of neuronal influence on the muscle-tendon unit was not possible [25,26,27,28]. 

Recent reports have implicated the protein titin as playing a crucial role in the passive tension developed in muscle tissue after stretching; titin is reported to bear most of the passive load in the muscle [25,26]. In particular, differences in the elasticity between heart and skeletal muscle are related to the isoform of titin expressed in the two tissues [26]. Recently, Thacker *et al.* noted that BoNT-A injection alters the collagen content of muscle, changes the expression of the protein titin, and finally results in fiber type switching from fast to slow twitch myosin heavy chain expression [29]. The authors concluded that these molecular mechanisms may contribute to BoNT-A’s effect on skeletal muscle passive mechanical properties [29]. Thacker *et al.* offer a plausible molecular explanation to some of the factors that may contribute to BoNT-A efficacious influence on stretching and physical therapy protocols [29]. However, differences in muscle elasticity cannot be attributed to titin alone. Although the stiffness in single spastic muscle fibers is increased compared to non-spastic muscle fibers [10] and the muscle cells in spastic muscles are shorter than in normal muscle tissue [30], the titin isoform expressed in healthy and spastic muscle cells are similar [27]. Another study showed that composite single muscle-fibers can have similar passive tension profiles and load-bearing protein composition, yet differences in passive tension profiles are still detected at the muscle bundle level when whole muscles are compared [28]. The present study has the advantage of examining passive properties in an *in vivo* setting of the intact muscle-tendon unit. The present study design has the limitation that identification of a specific molecular or structural mechanism by which BoNT-A influences passive biomechanical properties was not studied. However, the data demonstrate that the soft-tissue biomechanics are influenced by BoNT-A injection and the data are consistent with the structural changes observed by others and the proposed mechanisms by which passive properties are proposed to be molecularly modulated as previously published in the literature [10,27,28,29,30]. 

For the study of the physiological mechanism by which BoNT-A injections reduce *invivo* passive tension in the muscle-tendon unit, Protocol 3 was designed to evaluate differences in the recovery process after the muscle-tendon unit was stretched and returned to the initial length. The BoNT-A injected experimental group was found to exhibit a significantly reduced passive tension throughout recovery and a less robust recovery of the “resting passive tension”. The denervated muscle regained 15% of its initial resting tension, compared to a 30% recovery in the control, saline-injected muscle. We conclude that the adjunct BoNT-A injection modulates muscle tone, thus improving the ability of the muscle-tendon unit to stretch and to allow the muscle to return to the initial displacement length through chemical denervation. There are several other plausible mechanisms related to BoNT-A influencing the stretch reflex, which may, in conjunction with changes in muscle structure, contribute to its effect on muscle-tendon unit stretching.

**Figure 5:**
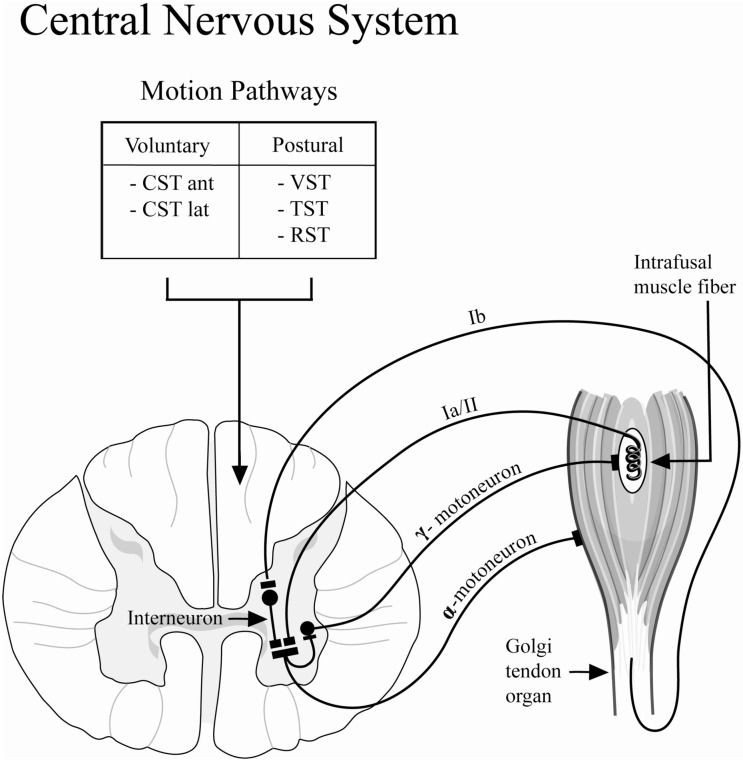
Schematic diagram depicting the stretch or myotatic reflex arc. The stretch reflex functions as a regulatory sensor for changes in muscle length and contributes to the resting tone of skeletal muscle. This reflex loop is modulated by the central nervous system through long-tract pathways: (1) Voluntary: corticospinal tract (CST): anterior (ant) and lateral (lat); (2) Postural: Vestibulospinal Tract (VST), Tectospinal Tract (TST), Rubrospinal Tract (RST).

The lengths of resting skeletal muscle and its response to changes in the length are regulated by the stretch reflex (Figure 5). The stretch reflex loop consists of the Golgi tendon organ, the intrafusal muscle fibers, afferent and efferent neural connections to the α- and γ-motoneurons, as well as inter-neurons. The intrafusal muscle fibers act to detect muscle length, and can adapt to changes in muscle length by causing responsive muscle contraction to restore the original length. This length adapting mechanism is controlled by the γ-motoneurons and their efferent innervation of the intrafusal muscle fibers. The γ-activation of the intrafusal muscle fibers utilizes the same neurotransmitter (acetylcholine) as the normal extrafusal muscle fibers; therefore, γ-motoneurons are susceptible to the inhibiting effect of BoNT-A [31]. Specifically, differences in the recovery of stretched muscles may result from the inhibition of the efferent arm of the γ-motoneuron. This inhibition would prevent the intrafusal muscle fibers from adapting to length change, rendering the length sensor for the muscle dysfunctional. This possible mechanism prevents the muscle from contracting to its original resting length after stretching exercises, thus, maintenance of resting tone or resting length may be impaired following BoNT-A injection.

A second mechanism thought to be involved in the reduced recovery tension after BoNT-A injection is related to the efferent arm of the α-motoneuron mediated reflex (Figure 5). The Golgi tendon organs sense stretching of the muscle-tendon unit and, in response, excite the α-motoneurons to activate muscle contraction in order to protect the muscle from damage due to excessive strain. Independent of intrafusal muscle fiber function, the efferent arm of the α-motoneurons is inhibited by the injection of the BoNT-A which acts through pre-synaptic inhibition of acetylcholine vesicular fusion and release into the neuromuscular junction, impairing synaptic activation of the muscle. Therefore, the percentage of tension initially produced at resting length is decreased in chemically denervated muscle due to pharmacological neuromuscular blockade of synaptic transmission resulting in decreased muscle contractility. 

A third mechanism by which BoNT-A injection may modulate passive *in vivo* muscle-tendon unit properties is related to the structure of the titin protein [25]. Titin has the ability to adapt its stiffness based upon the concentration of intramuscular cytosolic calcium [32]. Increased amounts of calcium in the cytosol following muscle contraction have been reported to lead to a stiffer configuration of titin, increasing the stiffness of the muscle up to 25% [33]. Following BoNT-A injection, muscle contractility decreases, producing less force. The concentration of intracellular calcium in BoNT-A denervated muscle is therefore reduced compared to the non-denervated control muscle. This reduced calcium concentration potentially leads to a less stiff titin configuration in the BoNT-A denervated muscle, contributing to decreased passive tension after the stretched muscle is returned to the initial displacement (Protocol 3). The quantification and relative contribution of the three possible mechanisms (α-inhibition, γ-inhibition, decreased Ca^2+ ^for titin) by which adjunct BoNT-A injection modulates the passive properties of the muscle-tendon unit will be the subject of future investigations.

There are limitations of this study, which utilized young, healthy mice. Although there are similarities between human and mouse GC muscle, the muscles differ in weight, size, and fiber type composition. Further, healthy muscle and spastic muscle differ in structure; thus, interpretation of our data in the context of spasticity is limited [34] and future studies are needed to assess BoNT-A injections in a spastic muscle model system. BoNT-A was injected intramuscularly, which can potentially cause damage to the muscle or the tendon due to incorrect injection technique. Only a single dose, volume, and post-injection time point was evaluated during the present study due to availability of BoNT-A. The dose and volume of BoNT-A were based on previous reports on BoNT-A injection of mouse GC muscle [17,20] and experimentation was conducted one week after injection, in order to provide maximal skeletal muscle paresis, with limited muscular changes. However, one week following the dose and volume of BoNT-A used in this report, an approximate 20% decrease in muscle mass can be expected [17], which needs to be taken into consideration when comparing passive biomechanical properties [35]. Therefore, the changes in the passive properties may not be attributed to the chemical denervation entirely. The histological and molecular changes have been described by others and were not subject of this investigation [29].

This study investigated the acute effects of stretching a GC muscle-tendon unit. The long term effects and benefits of stretching exercises for patients with spasticity remain unknown. Despite the positive effects of stretching on altering passive muscle properties, through modulation of excitability and active contraction [11], a recent report demonstrated no clinical differences after stretching intervention was implemented in the treatment of patients with neurological conditions [36]. Another limitation of our study is that only a single, relatively short period of stretching was studied. Clinically, physical therapy protocols often include repetitive stretching exercises performed over a longer periods of time. However, to evaluate the influence of adjunct BoNT-A therapy on stretching, a shorter experimental time period was sufficient to demonstrate differences between study groups. Future studies are required to study the influence of time from injection on the passive properties of the muscle-tendon unit. Further studies also are needed to assess the influence of various stretching protocols on passive muscle-tendon unit properties. Animals were anesthetized during testing, which has the potential to alter the passive properties of the muscle-tendon unit. However, experimentation conditions, including dose and duration of anesthesia were the same for both of the experimental groups. 

## 5. Conclusions

BoNT-A significantly influences muscle-tendon unit *in vivo* biomechanical properties. While BoNT-A injection has been shown to alter the molecular structure of skeletal muscle, the current study suggests an influence of BoNT-A on the stretch reflex as well (Figure 4) [11]. This study evaluated the effect of BoNT-A on the passive biomechanical properties of healthy muscle during stretching, which may provide a possible explanation of the clinical success of adjunct BoNT-A injections to stretching during physical therapy in patients with spasticity. However, the findings of this study will need to be confirmed in spastic animal models, in which the experimental system has inherent pathology of the myotatic or stretch reflex.

## References

[1] Lance J.W., Burke D. (1974). Mechanisms of spasticity. Arch. Phys. Med. Rehabil..

[2] Gracies J.M. (2005). Pathophysiology of spastic paresis. II: Emergence of muscle overactivity. Muscle Nerve.

[3] Nielsen J.B., Crone C., Hultborn H. (2007). The spinal pathophysiology of spasticity from a basic science point of view. Acta Physiol..

[4] Watkins C.L., Leathley M.J., Gregson J.M., Moore A.P., Smith T.L., Sharma A.K. (2002). Prevalence of spasticity post stroke. Clin. Rehabil..

[5] Seyler T.M., Smith B.P., Marker D.R., Ma J., Shen J., Smith T.L., Mont M.A., Kolaski K., Koman L.A. (2008). Botulinum neurotoxin as a therapeutic modality in orthopaedic surgery: More than twenty years of experience. J. Bone Joint Surg. Am..

[6] Hoare B.J., Wallen M.A., Imms C., Villanueva E., Rawicki H.B., Carey L. (2010). Botulinum toxin a as an adjunct to treatment in the management of the upper limb in children with spastic cerebral palsy (update). Cochrane Database Syst. Rev..

[7] Deon L.L., Gaebler-Spira D. (2010). Assessment and treatment of movement disorders in children with cerebral palsy. Orthop. Clin. North. Am..

[8] Koman L.A., Smith B.P., Shilt J.S. (2004). Cerebral palsy. Lancet.

[9] Unlu E., Cevikol A., Bal B., Gonen E., Celik O., Kose G. (2010). Multilevel botulinum toxin type a as a treatment for spasticity in children with cerebral palsy: A retrospective study. Clinics.

[10] Foran J.R., Steinman S., Barash I., Chambers H.G., Lieber R.L. (2005). Structural and mechanical alterations in spastic skeletal muscle. Dev. Med. Child. Neurol..

[11] Bovend’Eerdt T.J., Newman M., Barker K., Dawes H., Minelli C., Wade D.T. (2008). The effects of stretching in spasticity: A systematic review. Arch. Phys. Med. Rehabil..

[12] Bressel E., McNair P.J. (2002). The effect of prolonged static and cyclic stretching on ankle joint stiffness, torque relaxation, and gait in people with stroke. Phys. Ther..

[13] Hale L.A., Fritz V.U., Goodman M. (1995). Prolonged static muscle stretch reduces spasticity. S. Afr. J. Physiother..

[14] Yeh C.Y., Tsai K.H., Chen J.J. (2005). Effects of prolonged muscle stretching with constant torque or constant angle on hypertonic calf muscles. Arch. Phys. Med. Rehabil..

[15] Carey J.R. (1990). Manual stretch: Effect on finger movement control and force control in stroke subjects with spastic extrinsic finger flexor muscles. Arch. Phys. Med. Rehabil..

[16] De Jong L.D., Nieuwboer A., Aufdemkampe G. (2006). Contracture preventive positioning of the hemiplegic arm in subacute stroke patients: A pilot randomized controlled trial. Clin. Rehabil..

[17] Stone A.V., Ma J., Callahan M.F., Smith B.P., Garrett J.P., Smith T.L., Koman L.A. (2011). Dose- and volume dependent-response to intramuscular injection of botulinum neurotoxin-A optimizes muscle force decrement in mice. J. Orthop. Res..

[18] Dietz V. (2008). Studies on the spastic rat: An adequate model for human spastic movement disorder?. J. neurophysiol..

[19] Eaton M. (2003). Common animal models for spasticity and pain. J. Rehabil. Res. Dev..

[20] Stone A.V., Ma J., Whitlock P.W., Koman L.A., Smith T.L., Smith B.P., Callahan M.F. (2007). Effects of botox and neuronox on muscle force generation in mice. J. Orthop. Res..

[21] Mannava S., Callahan M.F., Trach S.M., Wiggins W.F., Smith B.P., Koman L.A., Smith T.L., Tuohy C.J. (2011). Chemical denervation with botulinum neurotoxin a improves the surgical manipulation of the muscle-tendon unit: An experimental study in an animal model. J. Hand Surg..

[22] Mannava S., Wiggins W.F., Saul K.R., Stitzel J.D., Smith B.P., Koman L.A., Smith T.L., Tuohy C.J. (2011). Contributions of neural tone to *in vivo* passive muscle-tendon unit biomechanical properties in a rat rotator cuff animal model. Ann. Biomed. Eng..

[23] Gajdosik R.L. (2001). Passive extensibility of skeletal muscle: Review of the literature with clinical implications. Clin. Biomech..

[24] Leonard T.R., Herzog W. (2010). Regulation of muscle force in the absence of actin-myosin-based cross-bridge interaction. Am. J. Physiol. Cell Physiol..

[25] Lieber R.L. (2002). Skeletal Muscle Structure, Function, & Plasticity: The Physiological Basis of Rehabilitation.

[26] Labeit S., Kolmerer B. (1995). Titins: Giant proteins in charge of muscle ultrastructure and elasticity. Science.

[27] Olsson M.C., Kruger M., Meyer L.H., Ahnlund L., Gransberg L., Linke W.A., Larsson L. (2006). Fibre type-specific increase in passive muscle tension in spinal cord-injured subjects with spasticity. J. Physiol..

[28] Silldorff M.D., Lane J.G., Lee K., Carr J.A., Gastwirt R.F., Lieber R.L., Ward S.R. (2011). Passive Mechanical Properties of the Human Supraspinatus and Infraspinatus Muscles. Proceedings of Annual Meeting of the Orthopaedic Research Society.

[29] Thacker B.E., Tomiya A., Hulst J.B., Suzuki K.P., Bremner S.N., Gastwirt R.F., Greaser M.L., Lieber R.L., Ward S.R. (2012). Passive mechanical properties and related proteins change with botulinum neurotoxin a injection of normal skeletal muscle. J. Orthop. Res..

[30] Friden J., Lieber R.L. (2003). Spastic muscle cells are shorter and stiffer than normal cells. Muscle Nerve.

[31] Stampacchia G., Bradaschia E., Rossi B. (2004). Change of stretch reflex threshold in spasticity: Effect of botulinum toxin injections. Arch. Ital. Biol..

[32] Labeit D., Watanabe K., Witt C., Fujita H., Wu Y., Lahmers S., Funck T., Labeit S., Granzier H. (2003). Calcium-dependent molecular spring elements in the giant protein titin. Proc. Natl. Acad. Sci. USA.

[33] Joumaa V., Rassier D.E., Leonard T.R., Herzog W. (2008). The origin of passive force enhancement in skeletal muscle. Am. J. Physiol. Cell Physiol..

[34] Lieber R.L., Steinman S., Barash I.A., Chambers H. (2004). Structural and functional changes in spastic skeletal muscle. Muscle Nerve.

[35] Frick C.G., Fink H., Blobner M., Martyn J. (2012). A single injection of botulinum toxin decreases the margin of safety of neurotransmission at local and distant sites. Anesth. Analg..

[36] Katalinic O.M., Harvey L.A., Herbert R.D. (2011). Effectiveness of stretch for the treatment and prevention of contractures in people with neurological conditions: A systematic review. Phys. Ther..

